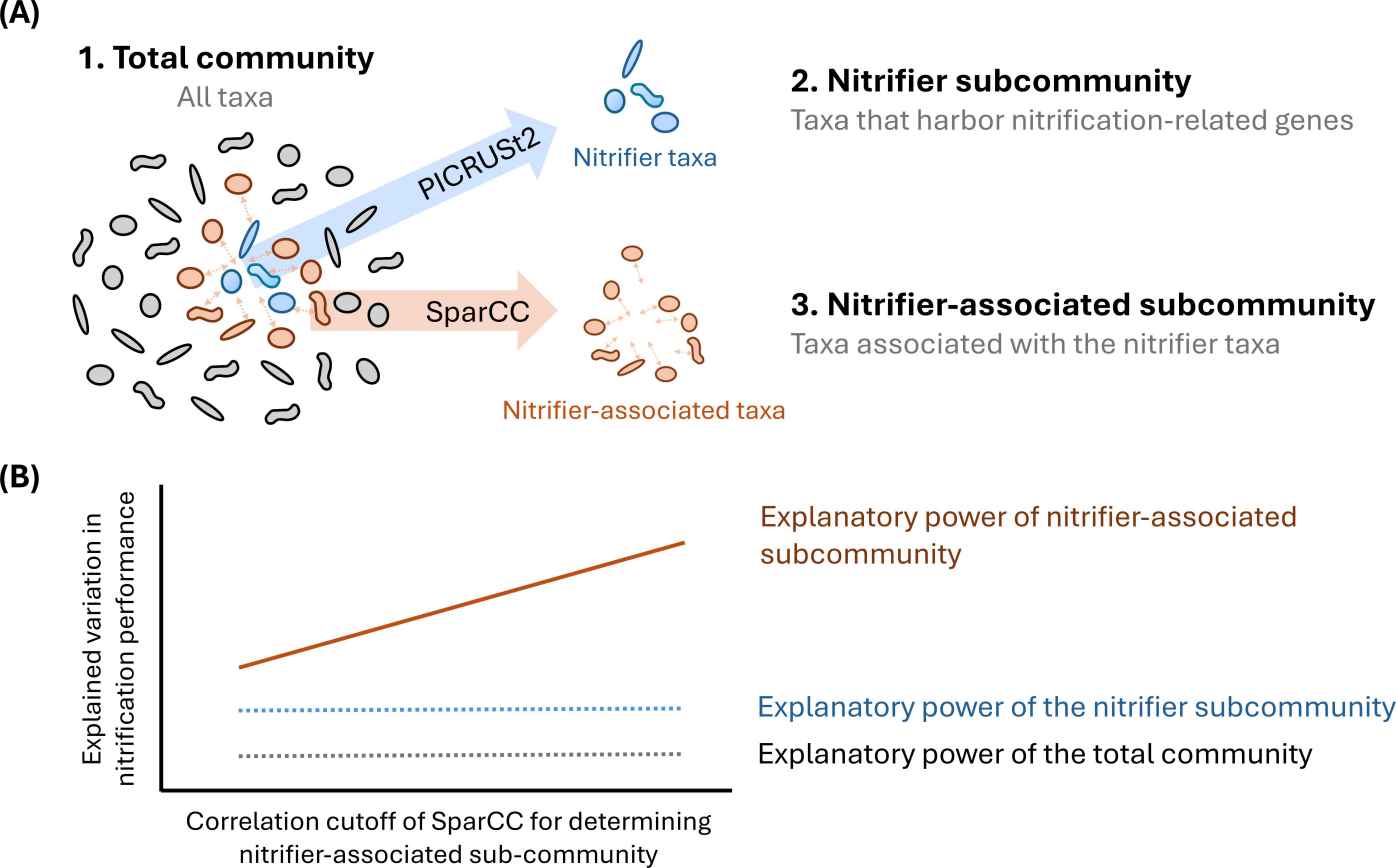# Articles of Significant Interest in This Issue

**DOI:** 10.1128/aem.02243-25

**Published:** 2025-11-19

## COMPOST TO THE RESCUE!

Compost is a valuable amendment for suppression of soil pathogens. Logo et al. (e01100-25) describe biomarkers of disease-suppressive composts, laying the groundwork for targeted isolation and functional studies of microbial-mediated suppression. 


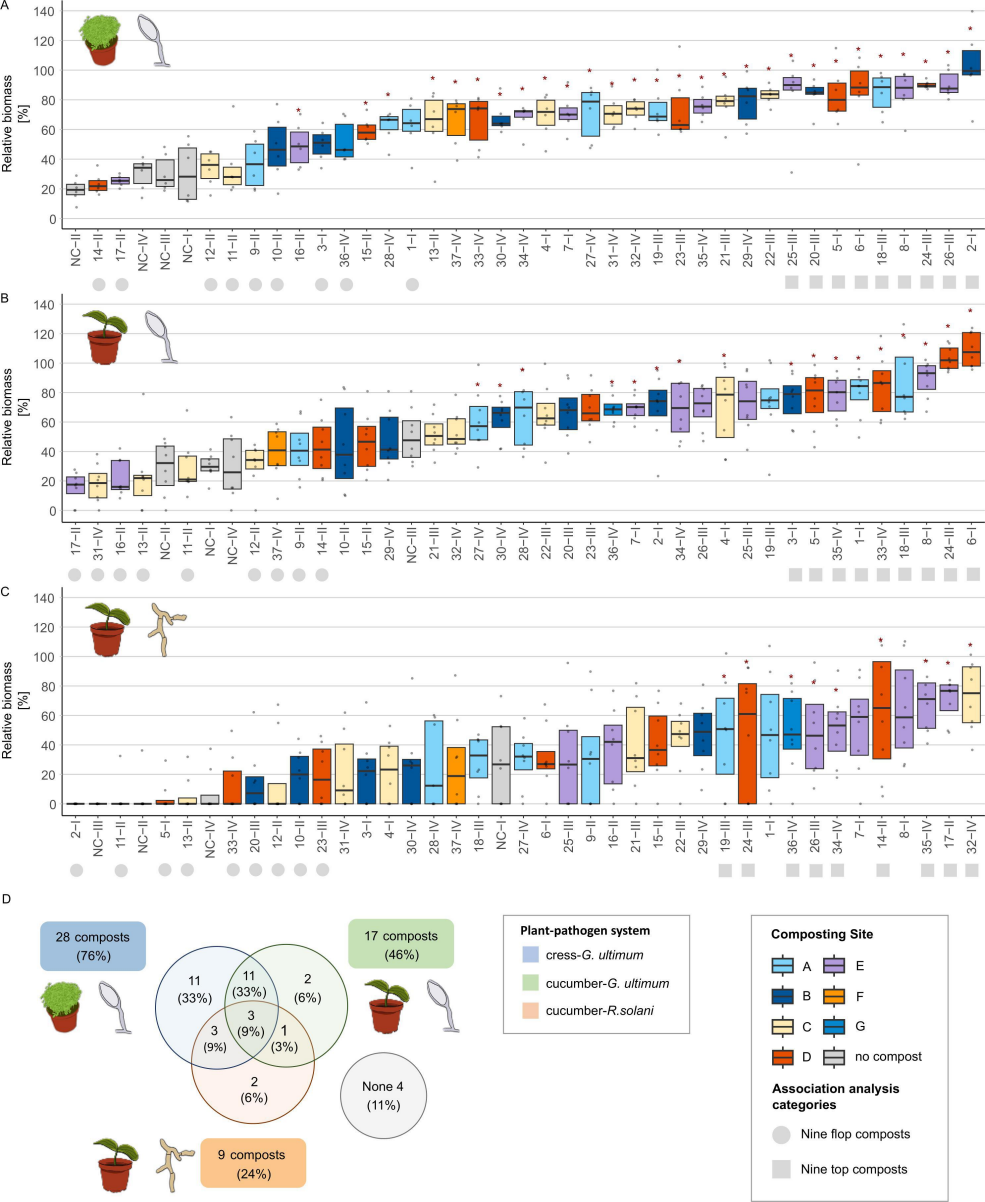


## A CLOSTRIDIAL APPROACH TO WEIGHT MANAGEMENT

This study by Meng et al. (e01152-25) reveals beneficial effects of *Clostridium butyricum* as a next-generation probiotic for gut microflora and weight management in mice. If scaled to humans, these findings promise novel approaches for anti-obesity nutritional products and pharmaceuticals.


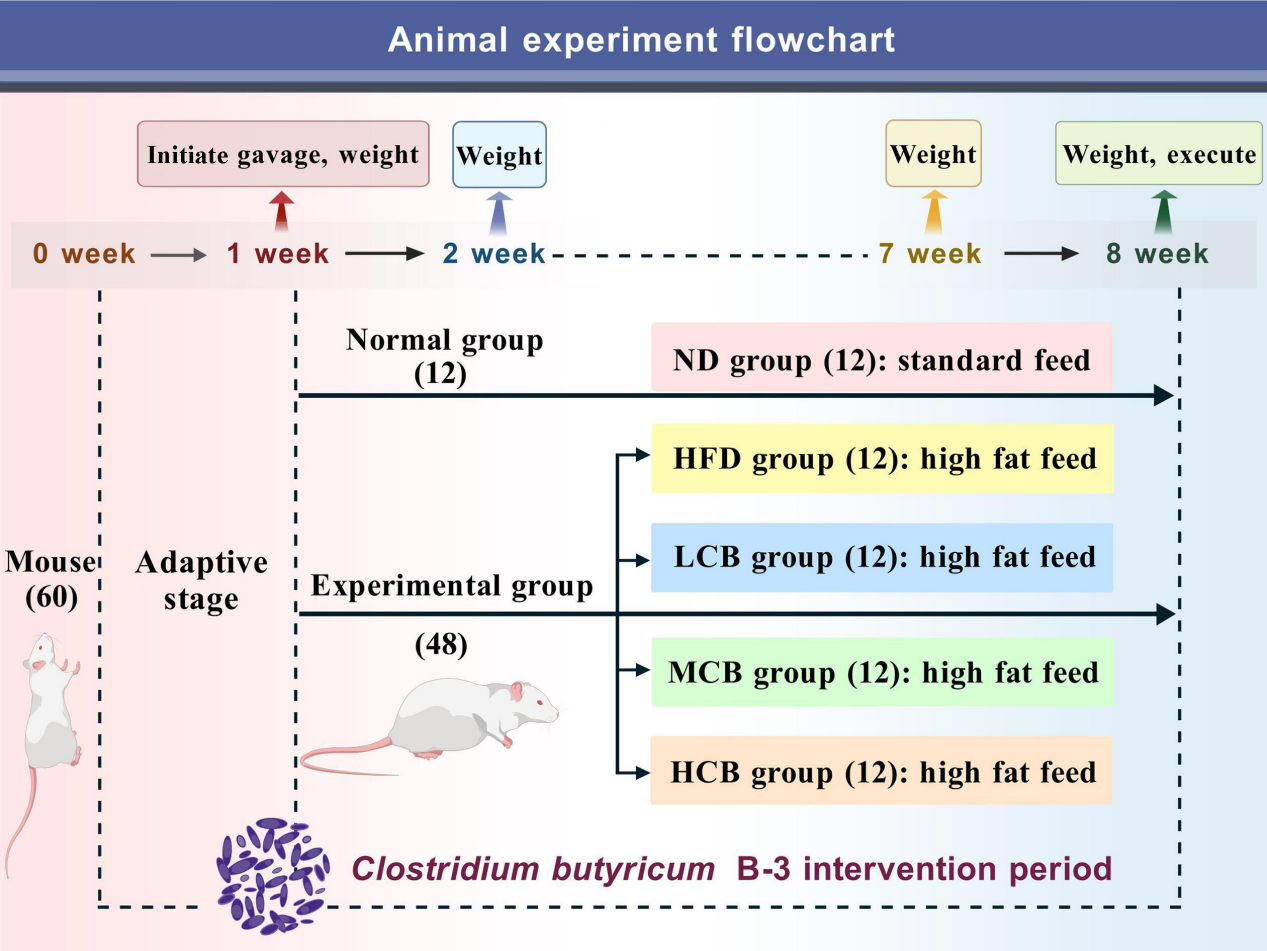


## MICROBIAL COMMUNITY RESPONSES TO FARMING MANAGEMENT

This commentary by Malak M. Tfaily (e01504-25) elegantly explains the significance of recent work published in *Applied and Environmental Microbiology* (AEM) (e00933-25) linking tillage legacy to microbiome-level adaptations critical to soil carbon management. A must-read to understand how farming management practices impact biogeochemical processes in terrestrial ecosystems.  


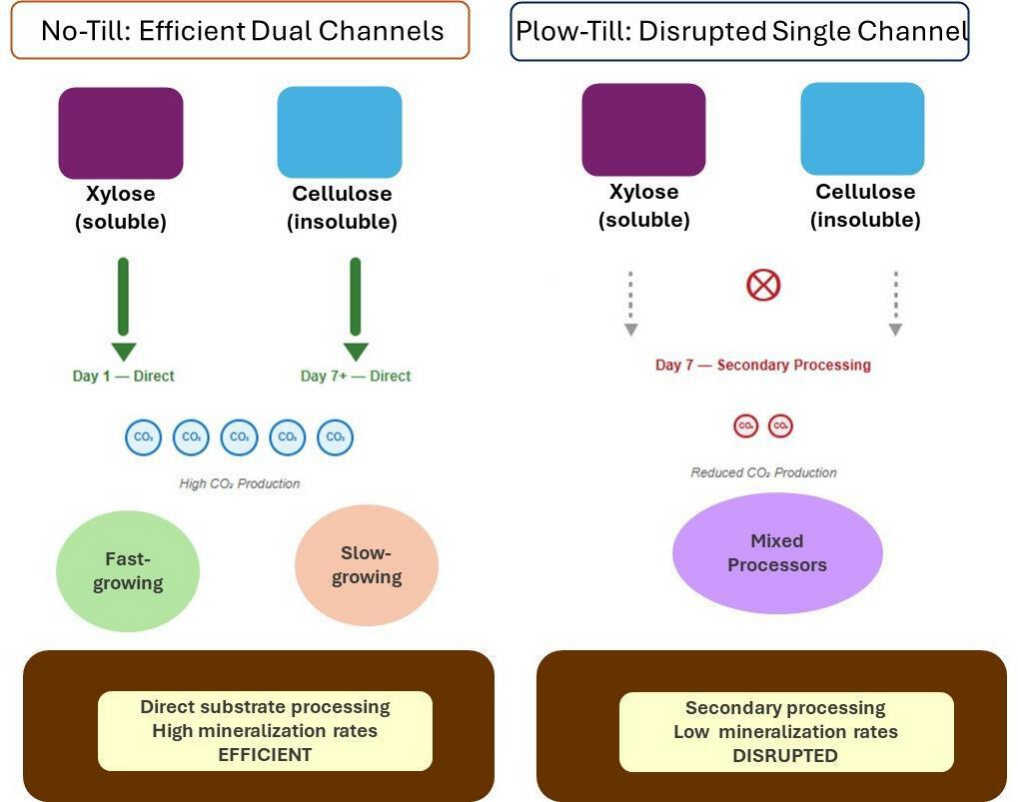


## TURNING FOES INTO FRIENDS

Finding antifouling strategies that are effective and environmentally safe remains a central challenge for maritime operations and ecosystem protection. This commentary by Raphaël Lami (e01609-25) builds on findings from a recent AEM study (e01392-25) to highlight the importance of microbial primers to build protective biofilm coatings against fouling. (*Image courtesy of Camille Ferré, Sorbonne Université, reprinted with permission*.) 


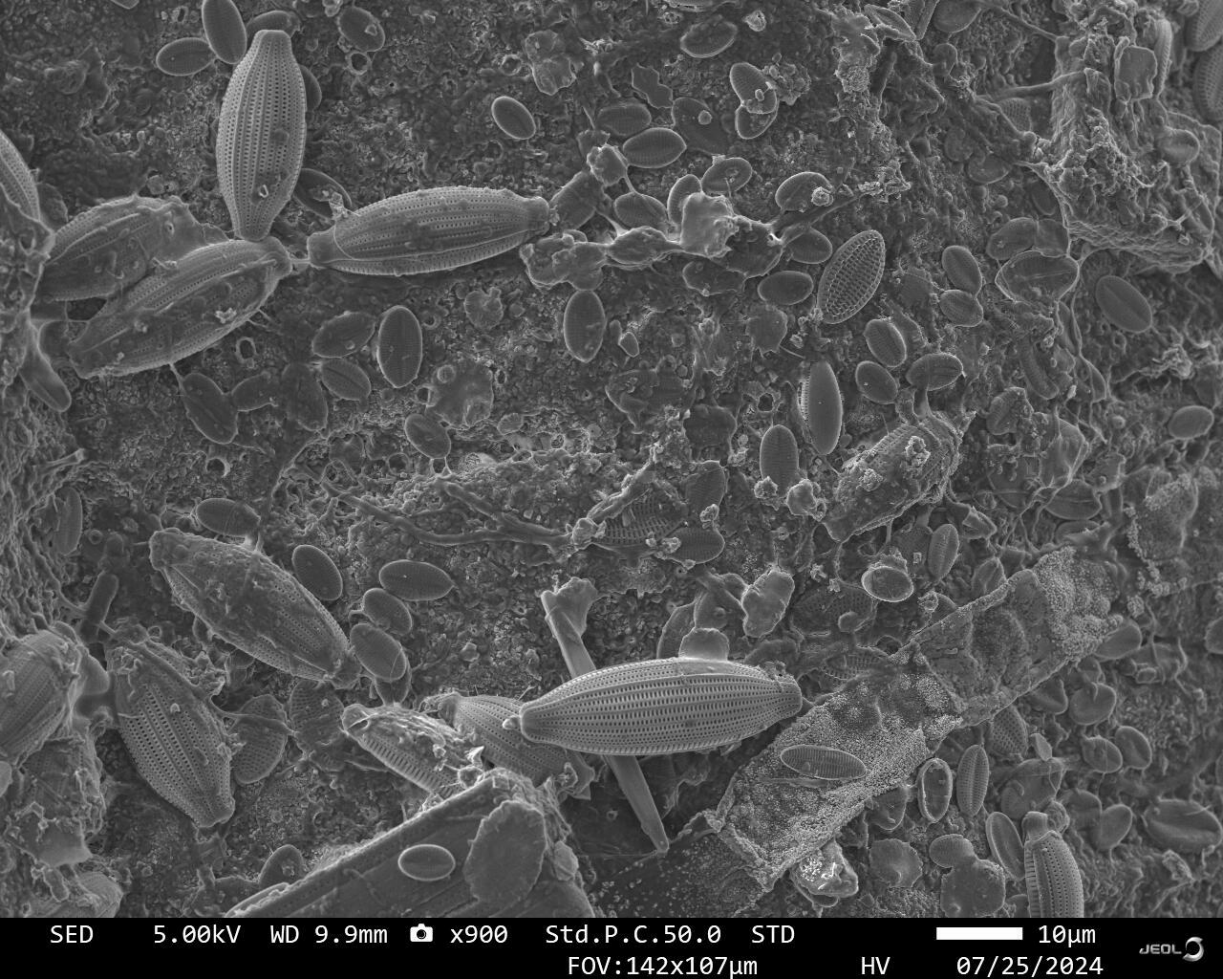


## PHAGE RESISTANCE À LA CRISPR

A study by Xu et al. (e01596-25) shows the benefits of CRISPR/Cas9 phage immunity for long-term, programmable protection of bacteria with minimal growth impairment compared to spontaneous mutations. The method can be applied to tailor solutions for mitigating phage contamination in industrial fermentations.


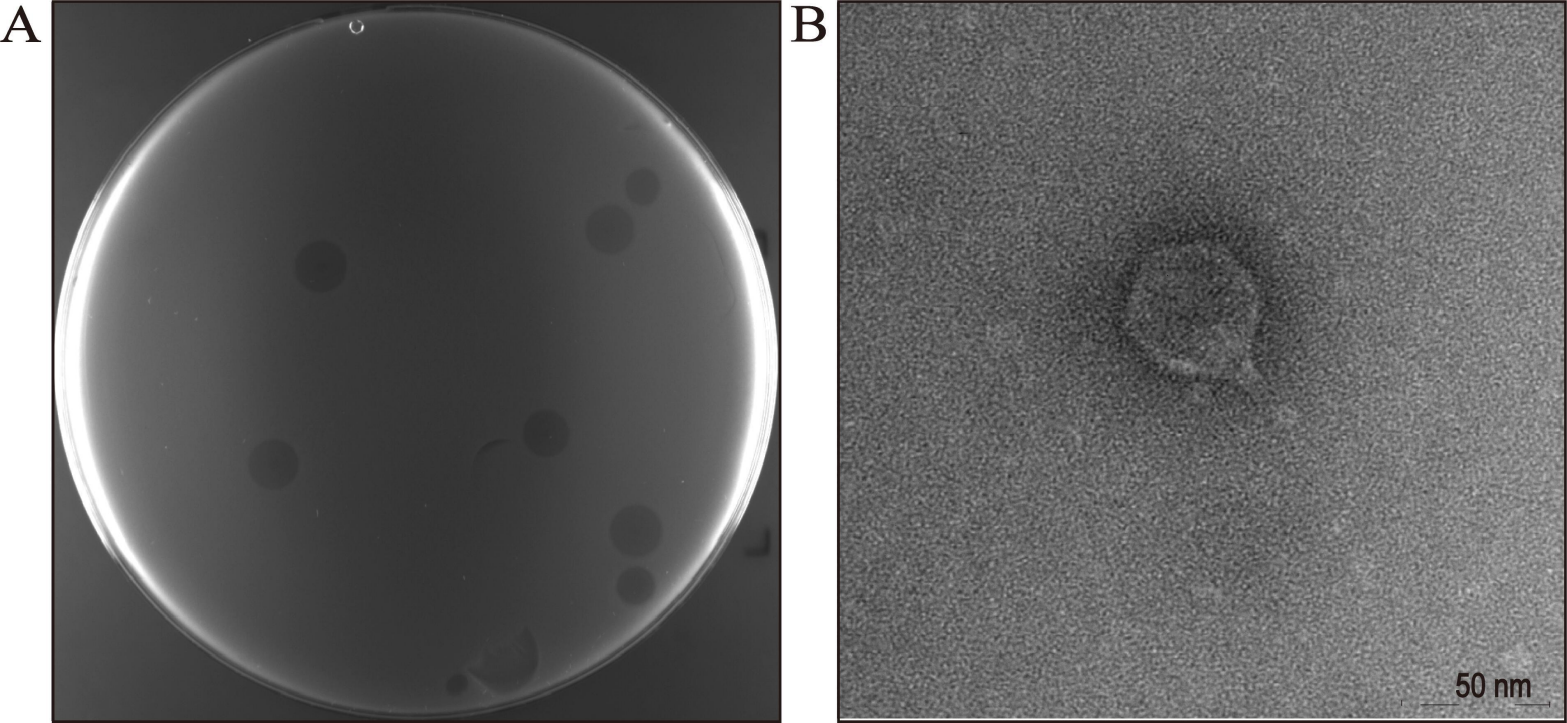
 

## THE CASE FOR MUCOFILMS 

This commentary by Mortensen et al. (e01269-25) recognizes mucofilms as distinct ecological microenvironments of host mucus and biofilm exopolymeric substances (EPS) controlling host-microbe-phage interactions, warranting further study of mucofilm towards improved therapeutics.


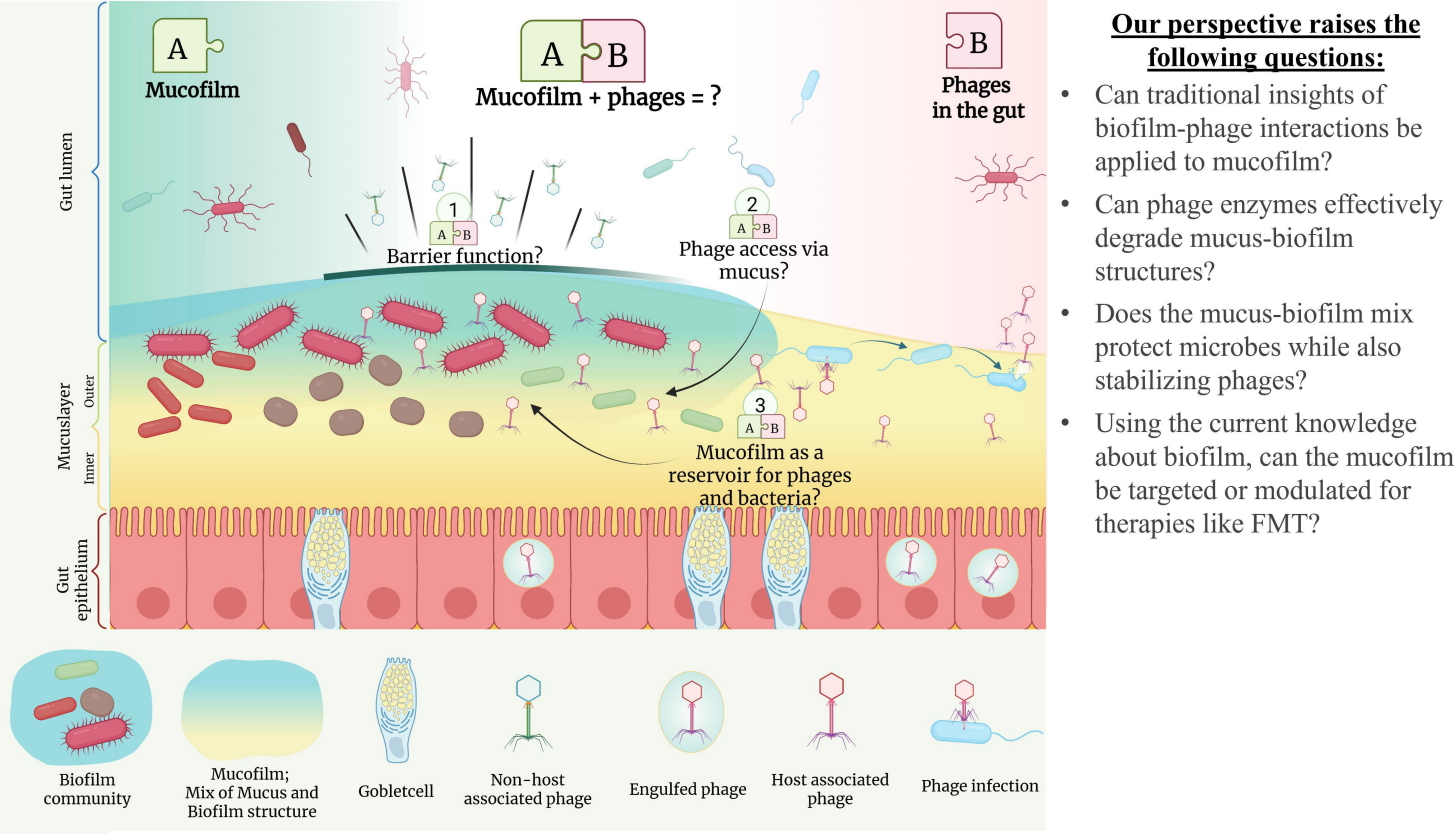


## IN THE COMPANY OF NITRIFIERS

Shao et al. (e01803-25) found that the diversity of microbes associated with nitrifiers, more so than the diversity of nitrifiers alone, impacts nitrification performance. This result highlights the importance of biodiversity in the management of nitrogen removal during wastewater treatment.